# Structural analysis of nursing quality issues in tertiary hospitals based on quality control inspections: a case study of 59 hospitals in Shanghai

**DOI:** 10.3389/fpubh.2025.1677639

**Published:** 2026-01-14

**Authors:** Yuhan Cheng, Yifan Jiang, Rui Tai, Lina Yang, Qin Xiang, Ying Wang, Yuli Liu, Yi Sheng, Yan Shi, Li Wang

**Affiliations:** 1 Shanghai Tenth People’s Hospital, School of Medicine, Tongji University, Shanghai, China; 2 Shanghai General Hospital, Shanghai, China; 3 Shanghai 6th Peoples Hospital Affiliated to Shanghai Jiao Tong University, Shanghai, China; 4 School of Nursing, Jinggangshan University, Ji'an, China; 5 Shanghai Yangzhi Rehabilitation Hospital, Shanghai, China

**Keywords:** health services management, nursing informatics, nursing quality control, qualitative research, standardization, structural challenges, tertiary hospitals

## Abstract

**Background:**

As global healthcare systems face increasing pressure from population aging and diversified patient needs, ensuring high-quality, standardized nursing services has become a strategic priority. However, persistent gaps in nursing quality control mechanisms hinder the delivery of efficient, patient-centered care, especially in large-scale hospital systems.

**Objective:**

This study aims to identify structural barriers in nursing quality control implementation and to provide evidence-based insights for improving nursing management and service effectiveness in tertiary hospitals.

**Methods:**

A qualitative study was conducted using data collected from nursing quality control inspections in 59 tertiary hospitals in Shanghai during the second half of 2024. A total of 46 experts from 23 professional groups participated. Data were analyzed using content analysis to extract recurring themes and sub-themes. The study was ethically approved by the Tenth People’s Hospital affiliated with Tongji University.

**Results:**

Three major themes and seven sub-themes were identified. The primary issues included: (1) inadequate standardization of nursing procedures and operational protocols; (2) insufficient professional competency and training of nursing staff; and (3) deficiencies in nursing informatization and quality improvement systems. Underlying factors included disjointed management frameworks, superficial capacity-building efforts, and fragmented digital infrastructure.

**Conclusion:**

The findings highlight significant structural contradictions between institutional frameworks and clinical execution of nursing quality control, which are not unique to the local setting but may reflect broader challenges in complex healthcare systems. Future research should incorporate quantitative outcome indicators to evaluate the real-world impact of nursing quality initiatives and inform the development of scalable, evidence-based strategies for enhancing nursing performance and ensuring patient safety.

**Clinical relevance:**

This study provides actionable insights for hospital administrators and nursing managers to optimize nursing quality control systems. By addressing procedural standardization, professional competency development, and informatics infrastructure, the findings offer a practical framework for improving nursing quality and patient safety in tertiary hospitals.

## 1 Introduction

With the continuous deepening of population aging and the increasing diversity of healthcare demands, nursing has become an increasingly vital component of China’s healthcare system. It plays a critical role in addressing aging-related challenges, improving healthcare quality, and promoting the realization of national health goals (1, 2). However, current studies reveal that nursing in China still faces multiple challenges. The relative shortage and uneven distribution of nursing human resources have led to a significant gap between the supply of nursing services and the growing needs of patients for diverse and personalized care (3). Moreover, in many healthcare institutions, nursing quality control remains at the level of data collection and reporting, lacking systematic analysis and evidence-based application of the obtained information. This limits the depth and substance of nursing services and constrains quality improvement efforts (4). In response, the Action Plan for Further Improvement of Nursing Services (2023–2025) (5) emphasizes the need to promote nursing services that are “patient-centered, clinically integrated, and community-oriented, “calling for coordinated efforts from multiple stakeholders to improve service quality, enhance the patient experience, and meet differentiated care needs.

Nursing quality control serves as a critical mechanism for ensuring high standards, efficiency, and consistency across the entire nursing service process through systematic and standardized supervision (6). Scientific and structured quality inspections can facilitate the standardization of care procedures and the homogenization of service delivery, thereby promoting continuous improvement and optimization of nursing quality to meet the demand for high-quality, personalized healthcare services (7). Based on field data collected during nursing quality control inspections conducted in tertiary hospitals across Shanghai in the second half of 2024, this study systematically analyzes and summarizes the issues identified by expert reviewers. The aim is to reveal the structural challenges currently facing nursing quality in tertiary hospitals and to provide evidence-based insights for improving nursing quality and optimizing nursing management strategies across healthcare institutions.

## 2 Materials and methods

### 2.1 General information

A purposive sampling method was employed to select 46 quality control experts from 23 expert groups affiliated with the Shanghai Nursing Quality Control Center in the second half of 2024. The expert groups were numbered from Z1 to Z23. The general information of the quality control experts is shown in Table 1.

**Table 1:** General information on nursing quality control inspector specialists (*N* = 46).

Group ID	ID	Professional title	Years of clinical experience	Years of nursing management experience	ID	Professional title	Years of clinical experience	Years of nursing management experience
Z1	N1	Chief Nurse	36	11	N24	Chief Nurse	29	10
Z2	N2	Chief Nurse	31	6	N25	Chief Nurse	41	17
Z3	N3	Chief Nurse	35	8	N26	Chief Nurse	37	12
Z4	N4	Chief Nurse	40	13	N27	Chief Nurse	49	17
Z5	N5	Chief Nurse	34	6	N28	Chief Nurse	37	11
Z6	N6	Chief Nurse	42	13	N29	Chief Nurse	25	9
Z7	N7	Chief Nurse	34	11	N30	Chief Nurse	37	10
Z8	N8	Chief Nurse	41	17	N31	Chief Nurse	38	12
Z9	N9	Chief Nurse	40	13	N32	Chief Nurse	30	8
Z10	N10	Chief Nurse	51	21	N33	Chief Nurse	41	13
Z11	N11	Chief Nurse	37	15	N34	Chief Nurse	31	8
Z12	N12	Chief Nurse	49	22	N35	Chief Nurse	28	6
Z13	N13	Chief Nurse	32	9	N36	Chief Nurse	33	11
Z14	N14	Chief Nurse	34	12	N37	Chief Nurse	36	10
Z15	N15	Chief Nurse	46	20	N38	Chief Nurse	34	11
Z16	N16	Chief Nurse	29	7	N39	Chief Nurse	37	14
Z17	N17	Chief Nurse	44	17	N40	Chief Nurse	35	9
Z18	N18	Chief Nurse	36	11	N41	Chief Nurse	29	11
Z19	N19	Chief Nurse	43	16	N42	Chief Nurse	40	15
Z20	N20	Chief Nurse	25	5	N43	Chief Nurse	35	11
Z21	N21	Chief Nurse	35	13	N44	Chief Nurse	36	13
Z22	N22	Chief Nurse	34	7	N45	Chief Nurse	32	9
Z23	N23	Chief Nurse	28	8	N46	Chief Nurse	39	12

### 2.2 Source of quality control data

The data originates from the nursing quality control inspection results conducted by the Shanghai Nursing Quality Control Center at 59 tertiary hospitals in Shanghai between September 10 and 25, 2024. The inspections followed a tracer-based management approach, with two-person expert teams composed of specialists from the municipal nursing quality control center, directors from district-level quality control centers, and members of the expert pool. These teams carried out on-site inspections, interviews, and investigations based on the actual nursing needs of patients, focusing on selected departments and frontline nurses. The duration for each hospital was determined by the time required to complete all evaluation items, with a minimum of half a working day (approximately 4 h). To ensure objectivity and minimize individual assessment bias, the two-person inspection teams were rotated across different hospitals; no single team visited all institutions, and team compositions were varied systematically throughout the inspection period.

The inspections were guided by the Evaluation Standards for Nursing Quality Control Inspections in Tertiary Hospitals (2024) issued by the Shanghai Nursing Quality Control Center. The evaluation covered hospital nursing management, ward management, tracking of key patients, implementation of the Action Plan (2023–2025), and the management of major diseases. It also assessed institutional standards for nursing ward rounds and shift handovers, human resource allocation, and problem rectification. Upon completion of each inspection, the identified issues were summarized and recorded in a standardized nursing quality control summary form. The data were then compiled and organized by the Shanghai Nursing Quality Control Center. This study was approved by the Ethics Committee of the Tenth People’s Hospital affiliated with Tongji University (Approval No. 22KN211).

### 2.3 Statistical methods

A conventional (inductive) qualitative content analysis approach (8) was adopted to analyze the existing nursing problems identified by expert teams across 59 tertiary hospitals. The analysis followed a structured, multi-stage process consisting of open coding, category formation, and theme abstraction.

Two researchers, both holding Master’s degrees in Nursing and possessing 3 and 8 years of clinical experience in tertiary hospitals respectively, conducted the analysis. Their combined expertise in both qualitative methods and clinical practice ensured the contextual validity of the coding. The coding process was managed using NVivo 12 software (QSR International) to facilitate data organization and retrieval. The analysis proceeded as follows: First, both researchers independently performed open coding on all textual materials line-by-line to identify recurring concepts. Codes were defined inductively based on semantic and contextual meaning. Through constant discussion and peer debriefing sessions, codes were compared and refined to ensure conceptual clarity, leading to the establishment of a preliminary coding framework. This framework was subsequently reviewed by a senior expert from the Shanghai Nursing Quality Control Center for content validity.

To test the reliability of the coding process, a subset of 20% of the data was double-coded independently, and inter-coder agreement was calculated using Cohen’s kappa coefficient, which reached 0.82, indicating high consistency. Discrepancies were resolved through discussion until full consensus was reached. Finally, codes were grouped into higher-level categories and abstracted into main themes and sub-themes. Representative examples were documented to illustrate how specific codes were applied, ensuring transparency and reproducibility of the analytic process.

To address potential sources of bias, such as variations in inspector rigor or the possibility of hospitals adjusting performance during inspections, the following measures were taken: inspection summaries were cross-validated for consistency, discrepancies were resolved through expert consensus, and all data were anonymized and aggregated at the institutional level.

## 3 Results

Based on the analysis of feedback from 46 nursing quality control experts, three major themes and seven sub-themes were identified. A total of 213 individual issues were identified from 59 hospitals based on expert feedback. Among them, 86 (40.4%) were related to insufficient standardization of nursing procedures and operational protocols, 74 (34.7%) concerned inadequate professional competency and training, and 53 (24.9%) involved deficiencies in nursing informatization and quality improvement systems.

In addition, 32 hospitals (54.2%) reported irregularities in nursing ward rounds and patient assessments, while 27 hospitals (45.8%) reported insufficient implementation of preventive measures for venous thromboembolism (VTE). Quantifying these issues highlights their prevalence and relative weight across different institutions and inspection teams, providing a clearer representation of the main areas of concern identified through the quality control inspections.

### 3.1 Theme 1: inadequate standardization of nursing procedures and operational protocols

#### 3.1.1 Irregularities in nursing ward rounds and patient assessment

The quality control of nursing ward rounds was found to be inadequate, with documentation content and formatting failing to meet required standards, and lacking targeted improvements for specialty-specific or critical issues.

Examples include: Z1: “The quality monitoring of nursing rounds was not effectively implemented.”; Z3: “Interdisciplinary ward rounds require further enhancement.”; Z12: “The ward round system is underdeveloped, lacking clear descriptions of purpose.”

Moreover, there was a general deficiency in patient assessment and nursing intervention competencies, particularly in the early identification and management of potential complications: Z2: “Poor predictive capability for complications in critically ill patients.” “Insufficient focus on perioperative condition changes and corresponding nursing priorities.”; Z9: “Inaccurate VTE risk assessments; nursing diagnoses and identification of potential problems were inadequately articulated.”; Z21: “Preventive strategies for VTE in key disease categories were not sufficiently detailed.”

#### 3.1.2 Poor implementation of nursing procedures and equipment/specimen management

Nursing procedures lacked timeliness and standardization, with weak implementation of responsibility-based nursing and high-quality practices: Z5: “Some operational protocols in the nursing unit were over 10 years old, with only initial dates provided and no evidence of review.”; Z12: “Surgical department consultation forms were overly simplistic; simulated scenarios for drainage care of surgical patients were poorly standardized.”; Z21: “Flexible endoscopes in the operating room were not transported in sealed containers, violating disinfection and isolation protocols.”

Disorganization was also observed in medical equipment management across wards and operating rooms, with insufficient standardization of material handling: Z4: “High-alert medications in emergency carts were not uniformly labeled; external-use water for injection and intravenous drugs lacked distinct markings.”; Z7: “Backup equipment storage in the ward was chaotic.”; Z21: “Assigned personnel for emergency item management were not effectively designated.”

### 3.2 Theme 2: inadequate professional competency and training of nursing staff

#### 3.2.1 Low proficiency in clinical skills and theoretical knowledge

There were significant individual differences in clinical competency among nursing staff, weak team collaboration, and an overall need to enhance skill levels: Z5: “Responses and operations regarding nursing standards for key diseases were incomplete.”; Z10: “Deficiencies in emergency response collaboration during urgent situations.”; Z21: “Lack of proficiency in defibrillator operation; poor implementation of complication management measures.”

Basic nursing knowledge was inadequately grasped, and specialized care capacity remained underdeveloped: Z6: “In key disease areas (e.g., gastrointestinal bleeding), responses on complication prevention were incomplete.”; Z7: “Common complications of clinical nursing techniques were not addressed, such as sudden coma and cardiac arrest.”; Z10: “The responsibilities and foundational training (‘three basics’) of nursing personnel at different levels were unclear.”

#### 3.2.2 Insufficient continuing education and professional training

There was a lack of institutional support for continuing education and specialty training, with limited scope and scheduling flexibility, hindering core competency development within nursing teams: Z3: “In some departments (e.g., operating rooms), in-service continuing education and teaching material development need improvement.”; Z4: “Tertiary training plans were unclear.”; Z7: “In the ‘three basics’ training, head nurses received only managerial training.”; Z13: “Mismatch between training content and initiation timing.”

There was also room for improvement in institutional development within nursing departments, particularly regarding policy clarity and the implementation of standardized group protocols:; Z13: “Specialty-sensitive indicators lacked standardization.”; Z15: “Group standards for common procedures—such as injections, VTE risk groups, and high-alert medication—were not properly implemented.”; Z17: “Institutional guidelines did not reflect re-evaluation timelines.”

#### 3.2.3 Uniform health education models and limited patient guidance approaches

Current clinical nursing practice predominantly adopts a generic and uniform model of health education, lacking disease-specific, tailored health promotion and patient guidance strategies. Health education efforts are insufficiently targeted and personalized, and nurses demonstrate weak capacity in delivering effective health education.

Examples include: Z4: “Primary nurses lack the ability to provide professional and individualized health education tailored to patient conditions.”; Z8: “On-site observation revealed a lack of specificity in nurses’ health education.”; Z10: “No guidance was provided regarding smoking and alcohol cessation.”; Z13: “Health education approaches lacked diversity, and preoperative patient instructions were insufficient.”

### 3.3 Theme 3: deficiencies in nursing quality control and informatization

#### 3.3.1 Insufficient development of nursing informatization and smart systems

Nursing management information systems are inadequately implemented, especially in areas such as rapid identification, reporting, and follow-up of adverse events.

Examples include: Z4: “The adverse event reporting hierarchy is not aligned with the current information system.”; Z10: “Lack of interoperability across information systems.”; Z6: “Clinical nursing informatics remains paper-based; nursing-related safety incidents are not reported via multiple channels and lack prompt response mechanisms.”; Z22: “Structured shift handover protocols need to be implemented in core systems; investment in nursing informatics should be strengthened.”

The overall development of informatics in nursing is clearly insufficient. Significant interdepartmental disparities exist in the application of digital tools, and smart technologies are often superficial without deep integration. Z6: “No implementation of ‘Internet + Nursing Services’ under the Action Plan.”; Z21: “Lack of mobile applications or follow-up systems for continuity of care; inadequate development of smart hospital, smart ward, and electronic medical record systems.”; Z23: “The hospital’s investment in nursing informatics infrastructure is inadequate.

#### 3.3.2 Inadequate implementation of nursing quality monitoring and improvement measures

Key nursing practices—such as ward rounds, drainage care for surgical patients, and specimen management protocols—suffer from poor standardization, leading to compromised service quality and hindering high-quality nursing development.

Examples include: Z2: “Routine quality monitoring in the ward lacks focus and does not reflect continuous quality improvement.”; Z5: “Ward-level policies, workflows, and protocols are less standardized compared to those of the nursing department.”; Z15: “The group standard on ‘Intraoperative Pressure Injury Prevention’ is not well integrated or enforced within operating room protocols.”

Efforts to improve nursing quality and safety are often hindered by a lack of data analysis and structural weaknesses in implementation, resulting in suboptimal quality monitoring outcomes: Z6: “No annual follow-up on adverse events in nursing management.”; Z12: “The Nursing Safety and Quality Management Committee lacks effectiveness; nurses are unfamiliar with annual adverse event trends, and feedback from the nursing department is insufficient.”; Z17: “No exemplary cases of nursing quality improvement; the organizational structure for quality management needs to be restructured.”

To enhance the transparency and traceability of the qualitative findings, a summary matrix was constructed to present the major themes, subthemes, and representative quotations extracted from the inspection data. This table provides a concise overview of the main problem categories identified during the analysis and illustrates how the qualitative evidence was coded and categorized across institutions (Table 2).

**Table 2:** Summary of major themes, subthemes, and representative quotations.

Theme	Subtheme	Representative quotation (Inspector ID)
Theme 1. Inadequate Standardization of Nursing Procedures and Operational Protocols	1.1 Irregularities in Nursing Ward Rounds and Patient Assessment	“Inaccurate VTE risk assessments; preventive strategies were not sufficiently detailed.” (Z9)
	1.2 Poor Implementation of Nursing Procedures and Equipment/Specimen Management	“Operational protocols were outdated and lacked regular review.” (Z5)
Theme 2. Inadequate Professional Competency and Training of Nursing Staff	2.1 Low Proficiency in Clinical Skills and Theoretical Knowledge	“Deficiencies in emergency response collaboration during urgent situations.” (Z10)
	2.2 Insufficient Continuing Education and Professional Training	“Mismatch between training content and initiation timing.” (Z13)
	2.3 Uniform Health Education Models and Limited Patient Guidance	“Health education lacked specificity and disease-oriented approaches.” (Z8)
Theme 3. Deficiencies in Nursing Quality Control and Informatization	3.1 Insufficient Development of Nursing Informatization and Smart Systems	“Adverse event reporting systems were not integrated with the hospital information platform.” (Z10)
	3.2 Inadequate Implementation of Nursing Quality Monitoring and Improvement Measures	“Routine quality monitoring lacked focus and continuous improvement.” (Z2)

## 4 Discussion

### 4.1 Structural contradictions in the operation of the nursing quality control system in Shanghai

The nursing quality control system in Shanghai demonstrates multidimensional structural contradictions in its implementation. Such contradictions between policy design and frontline practice are a central concern in implementation science, which examines the systemic barriers to integrating evidence into routine care (9). These contradictions are interrelated and hierarchical, collectively impeding the advancement of quality control from basic supervision toward a more refined and precise system. Although all 59 tertiary hospitals in Shanghai have established comprehensive institutional frameworks for nursing quality management, many hospitals have not developed detailed secondary and tertiary indicators under the primary supervision framework. Furthermore, insufficient differentiation between general and specialty nursing standards has led to a disconnect between institutional implementation and actual clinical practice.

Additionally, the lack of a well-structured nursing workforce and insufficient specialty service capacity have directly weakened the execution of quality control protocols. Studies show that the average bed-to-nurse ratio across the 59 tertiary hospitals in Shanghai is approximately 1:0.31 (±5.97), which falls significantly short of the national standard of no less than 1:0.65 as stipulated in the National Nursing Development Plan (2021–2025) (10). Moreover, there is a significant shortage of specialized nurses in geriatric care and chronic disease management (11). In the context of health education, most hospitals rarely adapt content based on patients’ age, education level, or lifestyle habits, making it difficult to meet patients’ needs for “precision-based” and “scenario-oriented” nursing services.

The lack of digital tools further exacerbates the gap between institutional enforcement and professional service delivery. In some hospitals, quality control inspections still rely heavily on manual documentation, with limited capacity for real-time data analysis and traceability, thereby compromising timeliness and accuracy.

### 4.2 Root causes of ineffective nursing quality control

The inadequate effectiveness of nursing quality control observed in practice is not an isolated issue, but rather the result of multiple overlapping structural contradictions.

First, the management system tends to exhibit “formalization” [often theorized as ‘symbolic compliance’ or ‘ceremonial conformity’ in organizational sociology (12)] rather than practical implementation, resulting in a disconnect between institutional frameworks and clinical practice. While some hospitals employ sensitive indicators for management purposes, the number of reported adverse events is often far lower than the actual occurrence rate, reflecting a preference for establishing systems over evaluating their real-world effectiveness. This form of “formalized” management reduces quality control to a procedural compliance exercise, failing to address the core issues of clinical care quality. It also fosters passive attitudes among frontline nurses who perceive quality inspections as superficial, further widening the gap between policy and practice.

Second, there is a “disruption” in professional development pathways. Capacity-building efforts remain superficial, with a lack of systematic training mechanisms (13). Training programs are often limited to basic theoretical and procedural competencies (“three basics”) and lack continuity and differentiation tailored to specialty nursing areas. Although most quality control personnel across the 59 tertiary hospitals in Shanghai hold undergraduate degrees, specialty-specific indicators remain insufficiently covered. Moreover, most hospital-level quality control team members are part-time, which limits their capacity to focus on the development of specialty competencies.

Third, the supporting infrastructure is weak. Informatization remains fragmented, lacking top-level planning and system integration. The disconnect between technological capabilities and the actual needs of quality control has created challenges. In some tertiary hospitals, quality control information systems are overly simplistic, plagued by data silos, and suffer from insufficient interdepartmental connectivity. These limitations exacerbate inefficiencies in nursing management and hinder data-driven decision-making processes (14).

### 4.3 Strategies for improving nursing quality control

To improve nursing quality and ensure patient safety, a comprehensive, multi-level strategy framework should be established, focusing on four key dimensions: resource integration, managerial reform, professional competency development, and technological support enhancement. Nursing quality improvement should be recognized as a core component of hospital strategic planning. Hospital leadership should prioritize quality enhancement and establish a hospital-wide Nursing Quality Management Committee, involving nursing, medical, and information departments to coordinate policy support, human resources, and nursing informatics development.

When viewed in an international context, the challenges observed in Shanghai’s tertiary hospitals—such as nursing workforce shortages, fragmented information systems, and limited use of data-driven quality monitoring—are consistent with patterns reported in other large healthcare systems (15). Studies from the United Kingdom, Japan, and Canada have similarly highlighted the structural tension between institutional accountability and frontline nursing execution, emphasizing the need for competency-based management and integrated informatics platforms (9). These parallels suggest that Shanghai’s experience reflects broader global trends, and that successful strategies from international nursing quality improvement programs could inform future policy design and local adaptation.

At the management level, outdated models that prioritize formality over substance should be abandoned in favor of outcome-oriented evaluation systems (16). Enhancing in-depth communication between quality control personnel and frontline nurses—shifting from a fault-finding to a collaborative coaching approach—can improve engagement and promote a shift from superficial compliance to intrinsic quality improvement (17).

Moreover, a structured and personalized competency development framework should be built. By conducting scientific competency assessments and designing individualized training plans based on nurses’ career stages and specialty needs, it is possible to significantly enhance clinical expertise and adaptability (18). Training methods such as virtual simulation and VR technology can further strengthen skills in specialty nursing, emergency procedures, and patient education, helping nurses internalize theoretical knowledge into routine clinical behaviors (19, 20).

Investment in nursing informatics is also crucial. Informatization can optimize care workflows, improve service efficiency, enhance the patient experience, and support evidence-based nursing management (21). Hospitals should adopt a strategic approach to informatics development by establishing unified data standards and shared platforms, enabling seamless integration of adverse event reporting systems, electronic health records, and smart nursing applications. Real-time data collection and analysis can facilitate dynamic quality monitoring, enable early identification of risks, and provide strong technical support for continuous nursing quality improvement (22).

The recommendations proposed in this study—including outcome-oriented evaluation systems, individualized competency frameworks, and investment in nursing informatics—are directly derived from the deficiencies identified through the inspection data. Specifically, the lack of measurable outcome indicators and fragmented data systems justify the call for outcome-based management, while gaps in specialty training and inconsistent skill development support the establishment of individualized competency models. Similarly, the limited integration of smart nursing systems highlights the necessity for enhanced informatics investment. Establishing explicit linkages between these findings and recommendations ensures that the proposed strategies are both evidence-driven and practically relevant to the identified structural issues.

## 5 Conclusion

This study conducted a comprehensive analysis of nursing quality control inspection data from 59 tertiary hospitals in 2024, identifying three major issues: inadequate standardization of nursing procedures, insufficient professional competency among nursing staff, and underdeveloped nursing informatics infrastructure. These findings reveal a broader disconnect between nursing quality control systems and their practical implementation at the clinical level—an issue that may extend beyond the local context to other large-scale healthcare systems.

Although this study offers meaningful insights into structural and operational gaps in nursing quality management, it remains limited by the absence of quantitative outcome indicators for evaluating the actual impact of quality control interventions. These structural insights translate into actionable recommendations for enhancing national and regional nursing policy. Specifically, to strengthen the implementation of national directives like the Action Plan for Further Improvement of Nursing Services (2023–2025), we recommend: (1) mandating the development and reporting of nurse-sensitive outcome indicators to shift evaluation from process compliance to results-oriented improvement; (2) allocating dedicated resources for competency-based, specialty career ladders to move beyond uniform basic training; and (3) integrating nursing informatics standards into broader health system digitalization plans to break down data silos and enable real-time quality monitoring. Future research should integrate nursing quality control findings with measurable care outcomes to facilitate data-driven evaluation and to establish a more generalizable framework for enhancing nursing quality and ensuring patient-centered, high-reliability care.

## Data Availability

The raw data supporting the conclusions of this article will be made available by the authors, without undue reservation.
